# Managing Antibiotic Shortages in Inpatient Care—A Review of Recent Years in Comparison with the Hungarian Status

**DOI:** 10.3390/antibiotics12121704

**Published:** 2023-12-07

**Authors:** Lilla Lőrinczy, Béla Turbucz, Balázs Hankó, Romána Zelkó

**Affiliations:** University Pharmacy Department of Pharmacy Administration, Semmelweis University, 1085 Budapest, Hungary; lorinczylilla@gmail.com (L.L.); hanko.balazs@pharma.semmelweis-univ.hu (B.H.)

**Keywords:** antibiotic shortages, impact, antibiotic stewardship, hospitals, inpatient

## Abstract

This study aimed to summarize the screened articles on antibiotic shortages, compare them with the Hungarian Health Authority database, and identify the overlapping substances in shortages and handling practices. A systematic analysis was conducted using the provided keywords to filter out appropriate studies and incorporate them into this review. The studies were searched in the following databases: Reaxys, PubMed, Ovid, ScienceDirect, and Embase. The search time interval was 2000–2023, with the following keywords used: “antibiotic”, “shortage”, and “in clinic”. The shortage data for Hungary were collected and integrated within the specified timeframe. This was achieved through a comprehensive screening method to ensure comparability between the data from the literature review and the database. Based on the comparison, we have identified two groups of ingredients, the overlapping and not-overlapping ingredients. The mitigation practices were also categorized and evaluated to recommend good shortage management practices for Hungarian decision-makers and healthcare professionals. Our key conclusion was to enhance a shortage risk-based approach, including the legislative, health authority, and healthcare professionals responsible for therapeutic protocol and procuring or producing the necessary product. A widely approved shortage risk-based framework should be created to mitigate the impacts, including communication protocols, individual therapy planning, compounding of magistral products, and antimicrobial stewardship programs. The most common mitigation strategy is the substitution with available alternatives, but besides, a good understanding and implementation of antimicrobial stewardship programs is also crucial.

## 1. Introduction

The shortage of antibiotics in hospitals around the world has been significant over the past 20 years, making it difficult for doctors and clinical pharmacists to work with them to prescribe therapy. Many antibiotics, whether injectable or oral, have been affected. There were antibiotics for which the shortage was temporary, but the list also included drugs that had not been available for several years, such as piperacillin–tazobactam.

There are several reasons for antimicrobial shortages. In most cases, supply disruptions are caused by manufacturing problems such as lack of financial incentives [1] withdrawal from the market [1], quality assurance problems [2], lack of a raw material or contamination [3], and supply disruptions due to an epidemic such as the COVID-19 epidemic in 2019–2021 [4,5]. Inappropriate and excessive prescribing and use, especially of broad-spectrum medicines [4,6], may also be a reason which may lead not only to drug shortages but also to a decrease in bacterial susceptibility and the development of antimicrobial resistance (AMR). Drug shortages can be caused when a drug is less profitable for its Marketing Authorization Holders, and therefore, production is reduced [5,7]. Substitution with existing alternatives is a good solution, but antimicrobial stewardship programs and trained pharmacists are also important. Antimicrobial stewardship (AS) programs are coordinated activities to develop and measure optimal antibiotic use, helping healthcare professionals to use the right agent, at the right dose, for the right time, and in the right way. These programs include, for example, the correct use of antibiotics [1,7,8,9], particularly the avoidance of excessive or frequent use of broad-spectrum antibiotics. This can also help to reduce shortages while reducing the development of resistance mechanisms [4]. Other strategies to mitigate shortages include close collaboration between doctors and pharmacists [4,5,6,8,9,10], exchanging policies between countries [5,6], effective forecasting systems [1,3,4,5,7,9,10], appropriate guidelines for substitution [3,4,8,10], and incentives for manufacturers [3,5,7]. These programs can provide guidance on antibiotic use and can also help to address shortages adequately.

Appropriate antimicrobial stewardship when the specific drug is in short supply is a real challenge for physicians and the pharmacists working with them, especially for those working at the clinics. In most cases, drug substitution could be the solution [2,3,4,7,8,9,10], but this has the potential to have negative consequences, especially if generic formulations of the active substance are not available or cannot be procured. These can include negative outcomes such as treatment failure or delayed cure and overuse of broad-spectrum antibiotics, which can be a precursor to the development of resistance. There is also concern that an alternative agent may be more harmful if it has a less favorable side-effect profile, dosage, and route of administration. All of this can be detrimental not only to the patient’s health but also to the patient’s cooperation [4].

As the shortage of anti-infective medicines has a great impact on the Hungarian pharmaceutical market, we decided to investigate the characteristics of ATC (Anatomical Therapeutic Chemical classification system) group “J” (Anti-infective for Systemic Use); however, we only focused on the antibiotic scope of the group [11]. This manuscript aims to summarize articles on antibiotic shortages in inpatient care worldwide and present possible solutions to alleviate the shortage after comparing the Hungarian antibiotics shortage situation with the data from the systematic literature review and identifying common areas or local specialties in the context of impacted substances. We have analyzed Hungarian antibiotic-related shortages based on the Health Authority database. What are the main differences, and where are the overlapping sections regarding active substances and the related therapeutic areas?

## 2. Results

All the examined studies (Table 1) show that there are negative consequences for both healthcare workers and patients when antibiotic shortages persist. Therefore, it is important that these shortages are resolved as quickly as possible. Substitution with suitable alternatives to the drug has been identified as a potential solution, but in addition, antimicrobial stewardship programs have an important role to play in helping to alleviate antibiotic shortages to a large extent, thus making patient care more efficient, and easier for colleagues working on the ward. Table 1 summarizes the handling solutions. 

In the study by Miljković et al., a survey of antibiotic shortages was carried out with the help of pharmacists working in European hospitals and found that several antibiotics such as amoxicillin, gentamicin, linezolid, meropenem, teicoplanin, piperacillin/tazobactam, tobramycin, cefepime, and cefamandole were among the shortage items between 2013 and 2020. To alleviate the shortages, most pharmacists used substitution as part of appropriate antimicrobial stewardship programs, depending on the availability of alternatives, although they found fault with the availability and timeliness of information in the shortage reporting systems. It was noted that it would be important to keep the shortage reporting IT software up to date and detailed, and to ensure good communication between health professionals [4].

In the study by Chigome A. K. et al., antibiotic shortages in public-sector hospitals in South Africa were assessed using electronic questionnaires over six months in 2018. The drugs most cited in stock-outs were benzylpenicillins and cephalosporins, while inadequate supply systems were blamed as the cause of the shortage. It was described that certain steps should be taken as part of the antimicrobial stewardship to address the shortage, such as the use of therapeutic exchange policies, increased communication with both stockists and prescribers, and participation in national monitoring programs [6].

The study by Hsueh K. et al. looked at meropenem, imipenem, and piperacillin–tazobactam shortages at Barnes-Jewish Hospital in the US in 2015. Antimicrobial stewardship programs were implemented to conserve antibiotics, which included some guidelines and changes in decision support and appropriate communication. Additional antimicrobial stewardship programs included prospective auditing, active inventory tracking and substitution with alternatives, even if the drug in shortage was currently available [8].

In a 2015 study, Barber et al. at the University of Mississippi Medical Center in the US examined the impact of piperacillin–tazobactam antibiotic shortages on meropenem consumption. Antimicrobial stewardship programs such as appropriate physician–pharmacist communication, targeted medication alerts, and changes in dosage or frequency of drug administration were used alongside intensive meropenem substitution to alleviate the shortage, but there was still a 111% increase in meropenem consumption [9].

In a study by Gundlapalli et al. in 2011, trimethoprim–sulfamethoxazole (i.v.), amikacin, and foscarnet shortages were studied in US hospitals using a questionnaire-based survey. The solution to reduce the shortage was primarily substitution, and the agents used to do this were trimethoprim–sulfamethoxazole (p.o.) and pentamidine. Although practitioners indicated that alternatives were often less effective or more toxic, it is worthwhile using additional antimicrobial stewardship programs in addition to substitution, such as improving doctor–pharmacist communication and timeliness of the pages and messages indicating shortages, so that the shortage is communicated more quickly to prescribers and treatment planners. It is also important that more information on alternatives is available and accessible so that colleagues in clinics do not have to spend a lot of time researching the literature [10].

In a study by Shafiq N. et al., a survey was conducted between 2020 and 2021 in hospitals in several countries, including India, the UK, Switzerland, and South Africa. Antibiotics on the shortage list included penicillin, cefazolin, piperacillin–tazobactam, and others. The study recommends several mitigation tactics, including organized, coordinated, and strengthened forecasting of drug shortages, cooperation and exchange of medicines between countries, greater financial incentives for manufacturing, development of generic medicines, faster and easier licensing, and promotion of local production of medicines [5].

The study by Hitoshi Honda et al. looked at cefazolin shortages in a medical center in Tokyo. In addition to substitution, antimicrobial stewardship programs have been used and suggested to mitigate shortages, such as optimizing prescribing in terms of duration of therapy and frequency of administration, encouraging a switch from intravenous to oral formulations as soon as possible, and making the transmission of information about shortages faster and more efficient, not least because logistical planning and regulation are essential for national access. It is also important that the professionals involved in each antimicrobial stewardship program receive appropriate, structured training to facilitate and improve their efforts to alleviate the shortage. Finally, manufacturers should be encouraged to stockpile the active substance or the raw material from which the active substance is synthesized, thereby increasing local production [7].

In the study by Griffith et al., antimicrobial shortages at Memorial Hospital in Chicago were tracked for 3 months in 2011. The shortages included foscarnet, streptomycin, gentamicin, and trimethoprim–sulfamethoxazole, all of which were involved in nationwide shortages. Another interesting finding of the research was that 2/3 of the drugs involved in the shortage were generic and only 1/3 were originator products. To reduce the shortages, antimicrobial stewardship programs were implemented, including appropriate communication, continuous monitoring of the FDA (Food and Drug Administration) and ASHP (American Society of Health-System Pharmacists) drug shortage websites, monitoring of local stock levels, and changes in drug therapy such as dosage dilution or shortening of treatment duration. Other efforts have included allowing imports into the US and reserving a particular antibiotic for patients whose pathogens have become resistant to other agents [1].

In a study conducted by Rocha A. F. B. et al. between 2017 and 2018, a maternity hospital in the city of Fortaleza investigated the lack of penicillin and its synthetic derivatives, which was a serious problem in the treatment of women and children with syphilis, the first-choice drug for this disease. Penicillin was conserved by substitution, in most cases with ceftriaxone and cefazolin or a combination of drugs, and only newborns who were symptomatic at birth, premature, or with other conditions were treated with penicillin [2].

Eventually, in a study by Nagano et al., cefazolin deficiency in Japan was examined at interrupted intervals from 2016 to 2020. They studied which parenteral drugs would be chosen as a result of the drug shortage and how the cost of these drugs would change over this period. Substitution with alternative drugs (ceftriaxone, cefotiam, ampicillin/sulbactam) was the most common option. Due to the low prices of ceftriaxone and cefotiam, there was no significant increase in costs, but consumption of these drugs was high. Other suggestions were made to alleviate the shortage, such as financial incentives for manufacturers, regular use of shortage forecasting programs, and publication of adequate information on the alternatives listed [3].

After a thorough analysis of the data pertaining to the Hungarian market, we can present the following key observations.

Based on the ten sampling dates, we categorized the substances into four groups based on their occurrence in the examined shortage list (Table 2). The four categories are the following (in brackets, we displayed the number of occurrences from 1 to 10). The scale of relevance:

- Most relevant (8–10);
- Moderately relevant (5–7);
- Less relevant (3–4);
- Not significant (1).

The first group includes the most relevant shortages, where deficiencies were recorded eight or more times in the sampling period out of ten. Active substances in this category are linezolid, lymecycline, and piperacillin/tazobactam. Within the moderately relevant category, active substances that appeared on deficiency lists between 5 and 7 times across all sampling points are classified, including benzylpenicillin, gentamicin, cefazolin, ceftriaxone, and sultamicillin. Substances with fewer instances of deficit (3–4 times) during the reviewed period are categorized as less relevant, such as tetracycline, vancomycin, and meropenem. Finally, ampicillin, bedaquiline, cefuroxime, and phenoxymethylpenicillin were categorized as not significant as they appeared on only one deficiency list out of the ten samplings.

Transitioning the emphasis from frequency (relevance scale) to duration (importance scale) highlights the heightened importance of the following active substances: benzylpenicillin, gentamicin, linezolid, lymecycline, tetracycline, and vancomycin. In Table 2, we have highlighted with bold letters the ingredients considered of high importance from the aspect of the duration of examined shortages. We have marked the average duration times (in days) in brackets in Table 2, next to all active ingredients. In case an ingredient appeared more than once in the sampling evaluation and at least in one case the duration could not be determined, we put ND in the brackets regardless of how many times it was undetermined in the evaluation. If we could calculate a clear average, we put the value in the brackets.

To highlight the therapeutic importance of the substances, we collected the main indications for each antibiotic in Table 3. To estimate the therapeutic importance, we used the WHO Model List of Essential Medicines—23rd, 2023 [12], and the WHO Access, Watch, Reserve (AWaRe) list [13] as primary sources. Nevertheless, regarding some ingredients, we included additional literature sources [14,15,16,17] to allocate the main indications for therapeutic classification in Table 3.

## 3. Materials and Methods

### 3.1. Systematic Literature Review

A systematic literature review has been conducted, aiming at summarizing studies on antimicrobial shortage. Five databases were searched: PubMed, Reaxys, Ovid, ScienceDirect and Embase. The search started on the twenty-seventh of September 2022, and the last run was on the fifteenth of July 2023. The search terms included antibiotic, shortage, and in clinic, while the time interval was given as 2000–2023. Original peer-reviewed research articles written in English were included.

**Figure 1:**
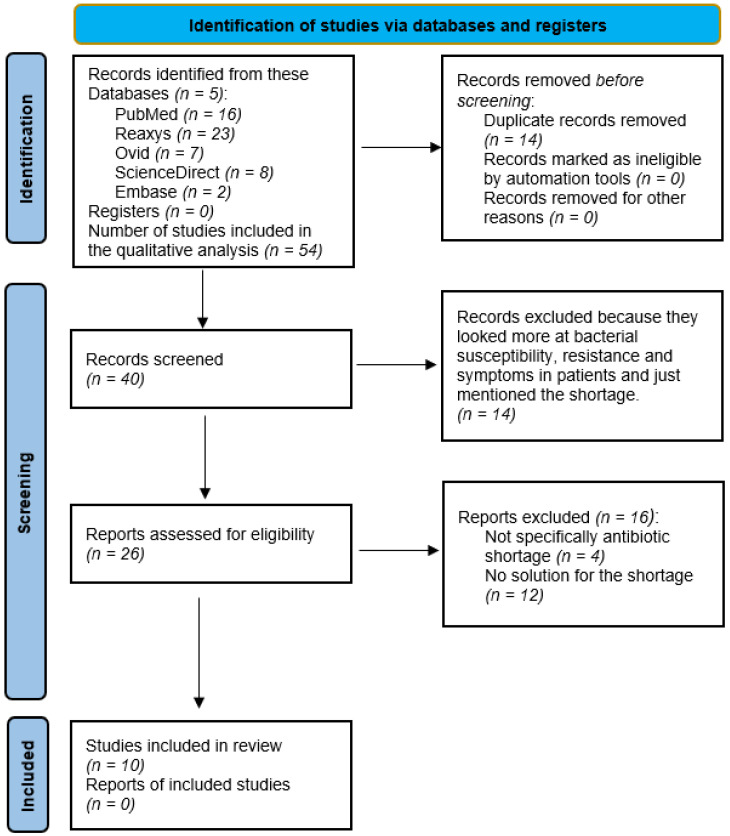
Flow diagram of the search [18].

The primary criterion for inclusion was that the topic should focus on antibiotic shortages. It was not sufficient to mention an existing shortage, but the article had to address the consequences of shortages, such as antimicrobial resistance and patient symptoms. Furthermore, the studies also had to present an adequate solution to the antibiotic shortage. In addition, the study should specifically address antibiotic shortage, it is not enough to just address a general drug shortage. Fifty-four articles were identified in the first round of analysis, and forty articles remained after removing duplicate descriptions. After further reading, another thirty articles had to be excluded. Of these thirty trials, fourteen looked at patients’ symptoms, bacterial susceptibility, and the development of resistance rather than addressing the shortage and were not included. Of the remaining twenty-six potential articles, we excluded four studies that did not specifically describe antibiotic shortages but other drug shortages, as well as twelve articles that described shortages but did not provide a possible solution. In the end, ten studies met all the criteria selected for the review, and a flowchart of the search is illustrated by Figure 1 [18].

We then looked at which inpatient care facility each study was conducted in and which and how many antibiotics were in short supply during the study period (shown in Table 4). The studies addressed the same issue but differed in several ways. Some studies looked at shortages occurring for a short period (less than half a year to a year), while others collected data on both drugs and solutions over several years. Furthermore, in some studies, only one type of antibiotic shortage was tested, while in others, a significant number of antibiotics were in shortage. As a final step in the systematic analysis, we summarized possible solutions to the antibiotic shortage based on the articles analyzed.

Overall, the selected studies had common objectives: to describe antibiotic shortages in different locations, to outline their negative consequences, and to describe different possible strategies for managing shortages.

### 3.2. Collecting Hungarian Antibiotic Shortages

Hungarian shortage data were collected and added after all the relevant articles were screened and analyzed. The relevant shortage data were downloaded from Comfit Europe Ltd.’s website, which is a company that monitors the information posted by the Health Authority and provides a processable database [19]. The database met all the necessary criteria, as it includes the active pharmaceutical ingredient, ATC code, the beginning of the shortage, expected end date, cause, and proposed action to mitigate the impact. We have examined the data published from 21 January 2019 to 20 June 2023. Instead of incorporating data dating back to 2000, we chose this timeframe due to alterations in the database’s properties in 2019. These changes enhanced its suitability for data filtering and comparison, as the characteristic of the database applied before 2019 only partially aligns with those of 2019 onwards. The data were sampled every six months; thus, ten databases were downloaded and filtered to include the above-described information.

To avoid the bias originating from the number of marketed presentations, which can be different country by country, we decided to focus on the impacted active ingredients, not the marketed presentations themselves; therefore, we performed ten queries by six-month frequency to compare the results based on the defined time interval during the period under review [20]. The following selection scheme was used during processing.

As a first step, the shortages where the Health Authority recommended one of the following actions to reduce the impact were filtered out [20]:

(1) The same medicine in a different dose of strength;
(2) The same medicine in different pharmaceutical forms;
(3) The same medicine in different packaging forms;
(4) Other medications were available for the same or similar indication;
(5) The same active substance was available in the same pharmaceutical form;
(6) The same active substance was available in another pharmaceutical form;
(7) Drug that was declared substitutable by the Health Authority was available (list of substitutes published on the website).

We filtered out the repeated active substances with a focus on the “J” ATC group (Anti-infective for Systemic Use). As the last refining step, we manually selected and omitted all the active substances, not antibiotics, e.g., antiviral agents, antifungals, vaccines, and other medications used to treat infections.

As a next step, we have calculated the estimated duration of the shortages from the information provided by the Health Authority [20]. If the expected end of the shortage was not determined, or the shortage should have been already resolved on the examination day, we considered the duration as not determined. Drug shortages were considered important from a duration perspective if the end of the shortage was not determined or we could quantify the expected end of the shortage; however, this duration was significantly longer than for the other active substances sampled.

Furthermore, to calculate the clinical importance of filtered ingredients, we compared them (Table 3) to the active substances on the WHO Model List of Essential Medicines [12] and the WHO Access, Watch, Reserve (AWaRe) list [13]. It is important to highlight that the WHO essential list was updated in 2023. Nevertheless, the AWaRe list is from 2021.

## 4. Discussion

The impact of antibiotic shortages on patients at the individual level can be severe and various [5]. As Hungary is also part of the global supply chain channels, we should continuously monitor and evaluate the Hungarian situation and the global environment in parallel as good practices can also originate from the global environment, which could support the Hungarian shortage situation. The systematic literature reviews could help Hungarian Healthcare Professionals discover good practices from other facilities to manage similar shortages. Our main goal was to compare the Hungarian antibiotics shortage situation with the global experience based on the systematic literature review and address recommendations. Do the antibiotics in the Hungarian antibiotic shortage situation overlap with those identified in the systematic literature review, and if so, which antibiotics overlap, and do they belong to a specific therapeutic group? Both scenarios can lead the authors to valuable conclusions, which can help to define future research directions.

Considering the three aspects—incidence frequency, duration of shortages, and therapeutic classification—it is apparent that linezolid, lymecycline, benzylpenicillin, gentamicin, and piperacillin/tazobactam pose a substantial risk to patients. We highlight the significance of the ingredients: Linezolid is listed in the Reserve category, but lymecycline and piperacillin/tazobactam are also included in the WHO AWaRe list’s Watch list. These five ingredients appeared several times (at least five times) within the sampling process (See Table 2).

Cefazolin and ceftriaxone shortage is also worthwhile; nevertheless, all the identified five shortage incidents that affected these two WHO essential substances had been resolved in 115 days in summary. In summary, the average resolution period for these two substances was 23 days for cefazolin (Access) and 9 days for ceftriaxone. Although this is not considered a critical duration in the context of the other examined substances, ceftriaxone is part of the Watch list, so shortages affecting it must be considered a priority even if the duration is not critical.

Sultamicillin belongs to this cohort, and the well-averaged resolution period was 41 days. Furthermore, sultamicillin is not considered a WHO essential substance and is also part of the Access list.

Tetracycline (Access) and vancomycin (Watch), both included in the WHO essential ingredients list, suggest their essentiality. However, they belong to the less significant group as tetracycline appeared only four times, and vancomycin only three times in the sampling evaluation. Nevertheless, it is important to mention that in the case of tetracycline, all shortage was with an undetermined end date; in the case of vancomycin, it was two times. Meropenem (Watch) shortage periods take 35 days on average, so we consider it a lower risk.

Ampicillin (Access), bedaquiline (NA), cefuroxime (Watch), and phenoxymethylpenicillin (Access) appeared once during our sampling evaluation; however, we consider it important to highlight that the bedaquiline and cefuroxime shortage end dates were also undetermined, which requires further monitoring and special attention in further analysis.

The above-described observation leads to the conclusion that during the four-year period (2019–2023) under review, at least half of the period, none of the marketed presentations containing the active ingredients in Table 3 were available to patients and healthcare professionals in Hungary. This finding is considered significant, as the WHO summary of therapeutic indications table (Table 3) shows that they are used to treat severe clinical conditions. Since no equivalent domestic alternatives were available in Hungary, the authors consider that the above-described insight is significant considering that the study included only those agents for which no domestic alternatives were available. According to the Health Authority proposal, mitigating the shortage impact is only possible by obtaining individual import permissions or contingent approvals from the Health Authority. This fact carries significance because it implies that Hungarian patients with potentially critical medical conditions, who may struggle with complex bureaucratic processes, cannot obtain these medicines on time from within the country. Instead, they must rely on foreign sources, which can be costlier, take significantly more time, create additional paperwork for healthcare providers, and potentially delay the implementation of their treatment plans, if available at all.

So far, we have reviewed the shortages of active substances affecting antibiotics during 2019–2023 in Hungary at six months of sampling, and we have calculated an estimated duration in Table 2. We also identified their therapeutic importance using the WHO essential and AWaRe lists (Table 3) and assessed the global environment based on specific criteria by performing a systematic literature analysis. In the following steps, we will examine the relationship between Hungary and the literature analysis global environment. After comparing the data, we divided the active substances into two main groups:

1. During the systematic literature analysis, the lack of active substance was registered elsewhere: (benzylpenicillin, cefazolin gentamicin, linezolid, meropenem, piperacillin–tazobactam)

It is important to remark that the benzylpenicillin shortage was identified in 2018 in South Africa, which is out of the investigated period in Hungary; however, we considered it as significant because these dates are in a tight row.

2. Not registered anywhere during the systematic literature analysis (ampicillin, bedaquiline, ceftriaxone, cefuroxime, lymecycline, phenoxymethylpenicillin, sultamicillin, tetracycline, vancomycin)

The literature review describes several appropriate shortage management strategies that have been implemented in inpatient units. This may provide good guidance for antibiotic shortage management in Hungary. It can be said that good communication between professionals is essential in the implementation of any shortage management strategy.

In general, a possible first step to mitigate the shortage is substitution with existing alternatives [2,3,4,7,8,9,10] In Hungarian hospitals, this is done according to the substitution guidelines and their step-by-step approach [20].

From the number and duration of shortages (Table 2), healthcare professionals can conclude the patient’s likelihood of completing the primarily selected therapy. These preliminary known factors should be incorporated as a risk into the therapeutic protocols during the treatment planning (age, comorbidities, allergic profile, resistance, concomitant medications), and if the risk of a shortage is high it is essential to review the patient’s profile to determine whether another product, maybe with another active ingredient, is also available to switch therapy (individual therapy planning). This measure would directly mitigate the impact of the shortage since the amount of use would be decreased by the individual planning, making it more likely to remain available during the whole therapy in patients who cannot be given other active substances. Furthermore, individual therapy planning can help to avoid switching of active ingredients in the middle of the therapy, reducing the risk of further resistant bacterial strains. In addition, a lot of discussed antimicrobial stewardship programs can assist healthcare specialists and are also widely practiced in Hungary.

It is also possible to apply specific international import or exchange policies. This method was reported in three articles [1,5,6] in the literature review, and this mitigation strategy is of great importance in Hungary. An important insight for domestic health professionals is to examine whether there is a chance to import the concerned medicine from a third country if it is available there in the same indication. It is also important to know which ingredients are generally in similar shortage worldwide because this rules out the possibility of importing these medicines from these countries. That is why it would be important to create, approve, and maintain a communication matrix protocol which defines who can be contacted in case of a particular shortage to procure foreign packs as soon as possible.

In some cases, the pure active ingredient is available on the market even if none of the marketed presentations are available for several reasons, e.g., packaging material shortage, regulatory implementation due date, discontinuation, etc. In Hungary, it is possible to create medicines in the clinical pharmacy and retail pharmacies called “Magistral medicinal products” or compounding. Magistral medicinal products are those that the pharmacist produces, packages, and dispenses in the pharmacy following the official Pharmacopoeia regulations in accordance with the principles set out in the resolution issued by the Counsic of Europe on the quality and safety assurance requirement for medicinal products prepared in pharmacies for the special needs of patients. If the pure active ingredient is available in the country, there is a potential solution in case each part of the supply chain participates together.

By establishing a risk-based framework, there is a possibility to enhance the expanded production of magistral medicinal products beyond the scope of Good Manufacturing Practice (GMP) regulations. This framework should involve the direct oversight of healthcare professionals from Health Authorities, specifically focused on the circumstances of the manufacturing steps, including analytical support and manufacturing validation, and the physicians who should also approve therapeutic protocols before approving these medicines. This risk-based framework should be well defined and regulate potential patient population (individual therapy planning) and production circumstances.

It must be admitted that producing medicinal products outside of GMP puts a higher risk on the final products; however, the absolute lack of a particular product can put a higher risk instead on patients. The authors recommend creating a pragmatic risk-based framework approved by the legislates, Health Authorities, and the healthcare professionals’ community representatives to determine the level of risk by patient population in particular risk.

Identifying whether a shortage situation is isolated to a specific country like Hungary or a more widespread issue involving multiple countries is valuable for several reasons. Future studies should investigate and research these reasons to provide further practices.

- Assessment of Global Impact: If a shortage is limited to one country, it may be due to specific local factors, such as regulatory issues, production problems, or supply chain disruptions unique to that region. However, if the same shortage issue is observed in multiple countries, it could signal a more significant global problem that requires attention.
- Identification of Common Causes: Analyzing shortages across multiple countries can help identify common root causes that transcend national boundaries. For example, shortages may be linked to a shortage of raw materials, increased demand due to infectious disease outbreaks, or regulatory challenges affecting multiple countries.
- Collaborative Solutions: When shortages are widespread, it becomes more critical for countries to collaborate on finding solutions. International cooperation can involve sharing best practices, coordinating supply chain efforts, and exploring alternative sources of essential medicines.
- Policy Development: Identifying common shortages across countries can lead to developing international policies and guidelines to prevent and mitigate such shortages in the future. These policies can help ensure a more stable and reliable supply of essential medications globally.
- Patient Care and Public Health: Shortages of critical medications can significantly impact patient care and public health, leading to delayed or inadequate treatment. Recognizing and addressing shortages globally can help protect patient health and safety.
- Market Dynamics: Studying shortages in multiple countries can provide insights into the pharmaceutical market’s dynamics and the supply chain’s vulnerabilities. This information can be used to develop strategies for improving the supply chain’s resilience.

## 5. Conclusions

The difficulty is in planning the right therapy while ensuring that antimicrobial resistance (AMR) does not increase excessively and that patients do not experience unpleasant side effects. As we have seen, substitution with alternatives is the general solution, but there is also a key role for antimicrobial stewardship programs, which includes monitoring effective shortage prediction programs, expanding and updating information on available alternatives, appropriate therapeutic regimens, and encouraging drug substitution policies, imports, and manufacturers. All of this requires good communication and collaboration between doctors and pharmacists, and appropriate informatics to make therapy as smooth, fast, and effective as possible.

Comparing the data proves to be a challenge due to variations in collection methods, sources, and timeframes. Yet, by examining Table 1 and Table 3, certain trends become apparent. After comparing the data from Hungary and the data from the institutions examined in the analysis, there are overlapping findings, and we can also identify gaps that are primarily specific to Hungary. The most obvious overlap identified in the case of the penicillin derivatives shortage of these ingredients affected all the investigated inpatient units in Hungary. Shortages of a specific ingredient are not occurring with the same consequences in the patients. Even if they have the same medical conditions, the shortage of the original ingredient or the use of an alternative can be various, e.g., drug interactions, allergic reactions, comorbidities, age, drug resistance). Subsequently, conscious and individual therapy planning is necessary based on the preliminary shortage situations of the desired active ingredient to avoid changes during the started therapy, thus also reducing the resistant strain occurrence and keeping the essential ingredient for patients to whom alternatives are not available due to their health conditions. Therefore, a more pragmatic and risk-based approach framework, including a previously approved and maintained communication protocol, should be created to differentiate and balance these therapeutical conditions. The importance of producing magistral products by a previously risk-based method approved by all competent parties would also be considerable for the decision-makers in the future.

## Figures and Tables

**Table 1:** Possible solutions to alleviate the shortage of antibiotics based on the studies searched. Used acronyms: i.v. for intravenous and p.o. for per os.

Shortage Product(s)	Pharmaceutical Formulation	Substitutability	Handling the Shortage	Reference
amoxicillin, gentamicin, linezolid, meropenem, teicoplanin, piperacillin/tazobactam, tobramycin, cefepime, cefamandole	i.v. p.o.: amoxicillin	substitutability depending on alternatives	Alternatives-dependent substitution and antibiotic stewardship programs, through shortage warning systems	[4]
benzylpenicillin, cephalosporins, erythromycin	i.v., erythromycin: p.o.	substitution was not the applied solution	Antibiotic stewardship programs, national monitoring programs (supply management), exchange policies	[6]
meropenem, imipenem, piperacillin/tazobactam	i.v.	cefepime	Antibiotic stewardship programs, including awareness campaigns, active inventory tracking, and audits	[8]
piperacillin/tazobactam	i.v.	meropenem	Substitution policies, antibiotic stewardship programs, including identification consultations, collaboration between pharmacists and doctors	[9]
trimethoprim–-sulfamethoxazole, amikacin, foscarnet	i.v.	trimethoprim–sulfamethoxazole (p.o.), pentamidine	Antibiotic stewardship programs, including general communication on drug shortages, electronic lists of alternatives	[10]
benzylpenicillin, cefazolin, piperacillin/tazobactam	i.v.	substitution was not the applied solution	Organized, coordinated, and strengthened foresight, facilitation of regulatory processes, local pharmaceutical production, importing	[5]
cefazolin	i.v.	ceftriaxone, ampicillin/sulbactam, vancomycin	Antibiotic stewardship programs, including optimizing current antimicrobial prescribing practices in hospitals, promoting the development of an action plan, a policy of offering financial incentives	[7]
foscarnet, streptomycin, isoniazid, clindamycin, gentamicin	i.v., p.o.: isoniazid, clindamycin	substitution was not the applied solution	Antibiotic stewardship programs, including tracking updated information, maintaining local inventories of critical antimicrobial stocks, importing	[1]
derivatives of penicillin	i.v.	ceftriaxone, cefazolin	Treatment with other therapeutic schemes, search for alternatives, use of ceftriaxone	[2]
cefazolin	i.v.	ceftriaxone, cefotiam, ampicillin/sulbactam	Antibiotic stewardship programs, trade promotion, national government involvement, improving forecasting systems	[3]

**Table 2:** The significance of the shortage level for each active substance is determined by its frequency of occurrence during the examined period, with average duration (in days) illustrated in brackets. **Bold letters:** important ingredients by duration, *Italic letters:* Not WHO essential.

Most Relevant (8–10)	Moderately Relevant (5–7)	Less Relevant (3–4)	Not Significant (1)
**linezolid (1286)**	**benzylpenicillin (ND)**	**tetracycline (ND)**	ampicillin (78)
***lymecycline (ND)***	**gentamicin (ND)**	**vancomycin (ND)**	bedaquiline (ND)
piperacillin/tazobactam (24)	cefazolin (23)	meropenem (35)	cefuroxime (ND)
-	ceftriaxone (9)	-	phenoxymethylpenicillin (119)
-	*sultamicillin (41)*	-	-

**Table 3:** Therapeutic importance of the investigated substances.

Substance	WHO Essential	WHO AWaRe	Main Indication
linezolid	Yes	Reserve	Bacterial pneumonia Skin and skin structure infections, vancomycin-resistant enterococcal (VRE) infections, including infections complicated by bacteraemia [14].
lymecycline	No	Watch	Acne and other susceptible bacterial infections [15].
piperacillin/tazobactam	Yes	Watch	Complicated intraabdominal infections (severe), high-risk febrile neutropenia, hospital-acquired pneumonia, necrotizing fasciitis [12].
benzylpenicillin	Yes	Access	Community-acquired pneumonia (severe), complicated severe acute malnutrition, sepsis in neonates and children, syphilis [12].
gentamicin	Yes	Access	Acute bacterial meningitis in neonates, community-acquired pneumonia (severe), complicated intraabdominal infections, complicated severe acute malnutrition, sepsis in neonates and children [12].
cefazolin	Yes	Access	Surgical prophylaxis [12].
ceftriaxone	Yes	Watch	Acute bacterial meningitis, community-acquired pneumonia (severe), complicated intraabdominal infections (mild to moderate), complicated intrabdominal infections (severe), endophthalmitis Enteric fever, gonorrhoea, hospital-acquired pneumonia, necrotizing fasciitis, pyelonephritis or prostatitis (severe) [12].
sultamicillin	No	Access	Infections of the respiratory tract, ears, nose, and throat, urinary tract, skin and soft tissues, obstetric and gynaecological infections, gonorrhoea [16].
tetracycline	Yes	Access	Bacterial blepharitis, bacterial conjunctivitis, bacterial keratitis—trachoma [12].
vancomycin	Yes	Watch	Endophthalmitis, necrotizing fasciitis [12].
meropenem	Yes	Watch	Acute bacterial meningitis in neonates, complicated intraabdominal infections (severe), high-risk febrile neutropenia [12].
ampicillin	Yes	Access	Community-acquired pneumonia (severe), complicated intraabdominal infections, complicated severe acute malnutrition, sepsis in neonates and children [12].
bedaquiline	Yes	-	Antituberculosis medicines [12].
cefuroxime	Yes	Watch	Lower respiratory tract infections (e.g., acute and chronic bronchitis and pneumonia); upper respiratory tract infections (e.g., ear, nose, and throat infections such as otitis media, sinusitis tonsillitis, and pharyngitis) [17].
phenoxymethylpenicillin	Yes	Access	Community-acquired pneumonia (mild to moderate) Pharyngitis, progressive apical dental abscess [12].

**Table 4:** Antibiotic shortages, duration, settings, and location of each study.

Type of Antibiotic	Interval of the Studies	Inpatient Unit and Settings	Reference
amoxicillin, gentamicin, linezolid, meropenem, teicoplanin, piperacillin/tazobactam, tobramycin, aztreonam, cefepime, cefamandole, ticarcillin	2013–2020	European hospitals; European Association of Hospital Pharmacists (EAHP) surveys	[4]
cloxacillin, benzathine, benzylpenicillin, erythromycin	In 2018 for half year	South African public-sector hospitals; Descriptive surveys and quantitative research approaches	[6]
meropenem, imipenem, piperacillin/tazobactam	2015–2016	Barnes-Jewish Hospital in St. Louis, Missouri, USA; Retrieving and analyzing data, creating models	[8]
effect of piperacillin–tazobactam deficiency on meropenem	In 2015 for half year	University of Mississippi Medical Center, Department of Medicine-Division of Infectious Diseases, Jackson, USA; Quality improvement retrospective review	[9]
Trimethoprim–sulfamethoxazole, amikacin, aztreonam, foscarnet	2011	US hospitals surveyed by the Infectious Diseases Society of America; By a nine-question survey	[10]
penicillin, cefazolin, clofazimine, dapsone, rifabutin, piperacillin–tazobactam, ceftolozane–tazobactam, cloxacillin	2020–2021	Some of the Hospitals from India, UK, South Africa, Switzerland; Database analysis	[5]
cefazolin	2019	Tokyo Metropolitan Tama Medical Center, Tokyo, Japan; Database analysis	[7]
amikacin, foscarnet, streptomycin, isoniazid, clindamycin, gentamicin and trimethoprim–sulfamethoxazole	2011	Northwestern Memorial Hospital (Chicago, IL); Prospective follow-up	[1]
amoxicillin, gentamicin, linezolid, meropenem, teicoplanin, piperacillin/tazobactam, tobramycin, aztreonam, cefepime, cefamandole, ticarcillin	2017–2018	Public maternity hospitals in the city of Fortaleza;	[2]
cefazolin	2016–2020	Japanese Hospitals; Database analysis	[3]

## Data Availability

Data are contained within the article.

## References

[1] Griffith M.M., Pentoney Z., Scheetz M.H. (2012). Antimicrobial Drug Shortages: A Crisis Amidst the Epidemic and the Need for Antimicrobial Stewardship Efforts to Lessen the Effects. Pharmacotherapy.

[2] Rocha A.F.B., Araújo M.A.L., Taylor M.M., Kara E.O., Broutet N.J.N. (2021). Treatment administered to newborns with congenital syphilis during a penicillin shortage in 2015, Fortaleza, Brazil. BMC Pediatr..

[3] Nagano H.S.J., Kunisawa S., Fushimi K., Nagao M., Imanaka Y. (2023). Impact of the cefazolin shortage on the selection and cost of parenteral antibiotics during the supply disruption period in Japan: A controlled interrupted time series analysis. J. Infect. Public Health.

[4] Miljković N., Polidori P., Kohl S. (2022). Managing antibiotic shortages: Lessons from EAHP and ECDC surveys. Eur. J. Hosp. Pharm..

[5] Shafiq N., Malhotra S., Pandey A.K., Holmes A., Mendelson M., Malpani R., Balasegaram M., Charani E. (2021). Shortage of essential antimicrobials: A major challenge to global health security. BMJ Glob. Health.

[6] Chigome A.K., Matlala M., Godman B., Meyer J.C. (2020). Availability and Use of Therapeutic Interchange Policies in Managing Antimicrobial Shortages among South African Public Sector Hospitals; Findings and Implications. Antibiotics.

[7] Honda H., Murakami S., Tokuda Y., Tagashira Y., Takamatsu A. (2020). Critical National Shortage of Cefazolin in Japan: Management Strategies. Clin. Infect. Dis..

[8] Hsueh K., Reyes M., Krekel T., Casabar E., Ritchie D.J., Jafarzadeh S.R., Hays A.J., Lane M.A., Durkin M.J. (2017). Effective Antibiotic Conservation by Emergency Antimicrobial Stewardship During a Drug Shortage. Infect. Control Hosp. Epidemiol..

[9] Barber K.E., Bell A.M., King S.T., Parham J.J., Stover K.R. (2016). Impact of piperacillin-tazobactam shortage on meropenem use: Implications for antimicrobial stewardship programs. Braz. J. Infect. Dis..

[10] Gundlapalli A.V., Beekmann S.E., Graham D.R., Polgreen P.M. (2013). Perspectives and concerns regarding antimicrobial agent shortages among infectious disease specialists. Diagn. Microbiol. Infect. Dis..

[11] Turbucz B., Major M., Zelko R., Hanko B. (2022). Proposal for Handling of Medicine Shortages Based on a Comparison of Retrospective Risk Analysis. Int. J. Environ. Res. Public Health.

[12] World Health Organization WHO Model Lists of Essential Medicines 23rd List 2023. https://www.who.int/publications/i/item/WHO-MHP-HPS-EML-2023.02.

[13] (2021). WHO Access, Watch, Reserve (AWaRe) Classification of Antibiotics for Evaluation and Monitoring of Use, 2021.

[14] Azzouz A., Preuss C.V. (2023). Linezolid. StatPearls [Internet].

[15] National Center for Biotechnology Information (2023). PubChem Compound Summary for CID 54707177. Lymecycline. https://pubchem.ncbi.nlm.nih.gov/compound/Lymecycline.

[16] Friedel H.A. (1989). Campoli-Richards. Sultamicillin. A review of its antibacterial activity, pharmacokinetic properties and therapeutic use. Drugs.

[17] Dellamonica P. (1994). Cefuroxime axetil. Int. J. Antimicrob. Agents.

[18] Page M.J., McKenzie J.E., Bossuyt P.M., Boutron I., Hoffmann T.C., Mulrow C.D., Shamseer L., Tetzlaff J.M., Akl E.A., Brennan S.E. (2021). The PRISMA 2020 statement: An updated guideline for reporting systematic reviews. BMJ.

[19] Comfit Europe LTDComfit Europe LTDComfit Europe LTDComfit Europe LTDComfit Europe LTDComfit Europe LTDComfit Europe, L.T.D. VigIntelligence Database Developed by VigIntelligence Kft. https://www.vigintelligence.com/Bejelentkezes.

[20] Nemzeti Népegészségügyi és Gyógyszerészeti Központ NNGYK Összes Termékhiány. https://ogyei.gov.hu/gyogyszeradatbazis.

